# What do you mean by “palliative sedation”? Pre-explicative analyses as preliminary steps towards better definitions

**DOI:** 10.1186/s12904-020-00635-9

**Published:** 2020-09-23

**Authors:** Alexander Kremling, Jan Schildmann

**Affiliations:** Institute of History and Ethics of Medicine, Interdisciplinary Center for Health Sciences, Madgeburger Straße 8, Halle (Saale), 06112 Germany

**Keywords:** Palliative care, Palliative sedation, Empirical research, Conceptual analysis, End-of-life care

## Abstract

**Background:**

Sedation in palliative care is frequently but controversially discussed. Heterogeneous definitions and conceptual confusion have been cited as contributing to different problems 1) relevant to empirical research, for example, inconsistent data about practice, the ‘data problem’, and 2) relevant for an ethically legitimate characterisation of the practice, the ‘problem of ethical pre-emption’. However, little is known about how exactly definitions differ, how they cause confusion and how this can be overcome.

**Method:**

Pre-explicative analyses: (A) systematic literature search for guidelines on sedation in palliative care and systematic decomposition of the definitions of the practice in these guidelines; (B) logical distinction of different ways through which the two problems reported might be caused by definitions; and (C) analysis of how content of the definitions contributes to the problems reported in these different ways.

**Results:**

29 guidelines from 14 countries were identified. Definitions differ significantly in both structure and content. We identified three ways in which definitions can cause the ‘data problem’ – 1) different definitions, 2) deviating implicit concepts, 3) disagreement about facts. We identified two ways to cause the problem of ethical pre-emption: 1) explicit or 2) implicit normativity. Decomposition of definitions linked to the distinguished ways of causing the conceptual problems shows how exactly single parts of definitions can cause the problems identified.

**Conclusion:**

Current challenges concerning empirical research on sedation in palliative care can be remediated partly by improved definitions in the future, if content and structure of the used definitions is chosen systematically. In addition, future research should bear in mind that there are distinct purposes of definitions. Regarding the ‘data problem’, improving definitions is possible in terms of supplementary information, checking for implicit understanding, systematic choice of definitional elements. ‘Ethical pre-emption’, in contrast, is a pseudo problem if definitions and the relationship of definitions and norms of good practice are understood correctly.

## Background

Sedation in palliative care is frequently and, simultaneously, controversially discussed, for example, regarding the indication, safety or justification compared to other end-of-life measures [1–4].

Good practice, research and fruitful discussion often depend on good and comprehensible terms. The problem with defining sedation practices and the existence of heterogeneous definitions has been criticised repeatedly in the light of associated problems relevant for good practice and research [5–8].

One problem which has been raised repeatedly in this context is that of inconsistent empirical data. It has been observed that there is an unexplained variation concerning the frequency of sedation in palliative care practice [9, p. 427], [10, p. 5], [11]. The vagueness of definitions and their variety are considered as contributing causes for this [9, 12], [13], [p. 2, 6].

In addition to this ‘data problem’, there is a second problem reported to be connected with definitions, which we call the ‘problem of ethical pre-emption’: Authors have argued [6, 14] that sedation is an ethically controversial procedure in palliative care. They criticise that moral discourse is decided beforehand (‘pre-empted’) when practices are defined as “therapy” or “ethically acceptable”, as it is the case in the well-known European Association for Palliative Care (EAPC) definition [15]. It has been suggested that exclusion of controversial cases of sedation by means of definitions produces a lack of transparency and open discussion and, consequently, a lack of a search for improvement [14].

While both the ‘data problem’ and the ‘problem of ethical pre-emption’ are alleged to have their roots in terminology, to the best of our knowledge, there has never been an analysis of how exactly definitions of ‘palliative sedation’ and similar terms cause these problems. In addition, there is a lack of rigorous conceptual analysis of existing definitions by which deficits could be identified and could pave the way for the improvement of definitions.

With this paper, we aim at supporting the comprehension of the terminological confusion, the resulting empirical and ethical problems and at developing strategies for creating clearer definitions that avoid the problems reported as much as possible. We tried to answer three questions to provide these analyses and tools for better definitions: (A) How exactly do definitions of sedation practices in palliative care differ? (B) How might definitions (logically) contribute to the data and ethical pre-emption problem? (C) How does definitional content contribute plausibly to the data or pre-emption problem regarding the ways identified?

## Methods

If any technical term is not shaped in an entirely novel way but explicitly also according to expressions that are already being used, then this process of term construction is called “explication” in theory of science [16–18] Given the heterogeneous and vague use of terms like “palliative sedation”, there is a need of such an explication.

It is standard to distinguish different steps in the explicatory procedure and to begin with preliminary steps in the literature on concept explication: discussing synonymy and ambiguity. These clarifications have also been called “pre-explicative procedures” [19]. Synonyms of “palliative sedation” and their pros and cons have been listed by others [6]. Our work focuses on the ambiguity problem by answering the three aforementioned questions (A)–(C). For this purpose we used different methods:

(A) To answer the question how definitions differ exactly, we conducted a systematic literature search (A 1) and extracted definitions and deconstructed them (A 2).

(A_1_) We focused on guideline definitions based on the assumptions that these have great impact and support in the respective community and have also been chosen thoughtfully. We conducted a systematic review following the PRISMA guideline [20] to identify relevant guidelines and searched the following databases: Medline, Cinahl, Embase, Psycinfo, Cochrane Library, Scopus and Web of Science up to 11 December 2019 (for Medline search string see Table 1). Additionally, we performed a web search via Google with a standardised search string and a search on selected websites for international medical literature and guidelines (Tripdatabase, Gin, Epistemonikos, AHRQ, Nice). Search terms were in English. There were no restrictions on publication language. We screened the reference lists of guidelines included and identified systematic reviews for other documents. We screened the first 200 search results when it was not possible to use a search syntax. We included national, regional or institutional guidelines with a focus on sedation in palliative care. We then excluded publications without institutional or regional/national legitimization and those with a broader focus (i.e. intensive care, end-of-life care in general). We searched for updated versions on the institutions’ websites and only included the latest versions. We included only original documents or our own translations when possible. We excluded publications with a broader focus (i.e. intensive care, end-of-life care in general). Each screening and data extraction was performed by the main author and a researcher assistant individually. Regarding the publications included, we extracted definitions and translated them if necessary, assisted by at least one native-speaking colleague with a medical background. We did not perform a formal quality assessment of the guidelines included, for example, with AGREE II [21].

**Table 1:** Search String (Medline)

	Search string
Medline	(Clinical pathway[mh] OR Clinical protocol[mh] OR Consensus[mh] OR Consensus development conferences as topic[mh] OR Critical pathways[mh] OR Guidelines as topic [Mesh:NoExp] OR Practice guidelines as topic[mh] OR Health planning guidelines[mh] OR guideline[pt] OR practice guideline[pt] OR consensus development conference[pt] OR consensus development conference, NIH[pt] OR position statement*[tiab] OR policy statement*[tiab] OR practice parameter*[tiab] OR best practice*[tiab] OR standards[ti] OR guideline[ti] OR guidelines[ti]
	OR ((practice[tiab] OR treatment*[tiab]) AND guideline*[tiab])
	OR CPG[tiab] OR CPGs[tiab] OR consensus*[tiab]
	OR ((critical[tiab] OR clinical[tiab] OR practice[tiab]) AND (path[tiab] OR paths[tiab] OR pathway[tiab] OR pathways[tiab] OR protocol*[tiab]))
	OR recommendat*[ti]
	OR (care[tiab] AND (standard[tiab] OR path[tiab] OR paths[tiab] OR pathway[tiab] OR pathways[tiab] OR map[tiab] OR maps[tiab] OR plan[tiab] OR plans[tiab]))
	OR (algorithm*[tiab] AND (screening[tiab] OR examination[tiab] OR test[tiab] OR tested[tiab] OR testing[tiab] OR assessment*[tiab] OR diagnosis[tiab] OR diagnoses[tiab] OR diagnosed[tiab] OR diagnosing[tiab]))
	OR (algorithm*[tiab] AND (pharmacotherap*[tiab] OR chemotherap*[tiab] OR chemotreatment*[tiab] OR therap*[tiab] OR treatment*[tiab] OR intervention*[tiab])))
	AND (“palliative sedation” OR deep sedation[mesh] OR (sedation AND (palliative care[mesh] OR palliative medicine[mesh] OR end-of-life OR eol OR terminal care[mesh] OR terminally ill[mesh] OR critical care[mesh])))

(A_2_) We then deconstructed the definitions. Sedation in palliative care is a medical practice. As there is no standard for definitional elements of practices, we used the following analytical categories to deconstruct the definitions: action, means, object, purpose, intended path of action, unintended consequence (see Table 2). These categories are influenced by the 20th century philosophy of action, for example [22], and are applicable to all practices on a very basic level.

**Table 2:** Categories for analysis of elements of sedation definitions

Category	Description	Medical example
Action	The activity described as directly executed by an agent	Administration of medications
Means	Artefacts or natural objects “with which” the action was carried out	Pharmaceuticals
Object	Things or people that necessarily undergo change if the action takes place	Terminally ill patients
Purpose	The event or state of affairs aimed at that is the main requirement for success of the action	Treatment of intolerable symptoms
Intended path of action	Event or state of affairs aimed at to achieve the purpose	Reduction of consciousness
Unintended consequence	Event or state of affairs which is not intended	No intention to end the patient’s life
Moral property	Qualifying expressions indicating positive or negative ethical evaluation	Ethically acceptable to the family

The category “explicit moral property” was added to the categories to display where the “normativity” criticised in the argument against ‘ethical pre-emption’ was explicitly present in the definition. We tested categories until we achieved “analytical saturation”, in the sense that definitions could be fully deconstructed. More fine-grained categories are possible but can be seen as subcategories of the ones we used.

(B) Answering the question how exactly definitions cause the ‘data problem’ and the ‘problem of ethical pre-emption’ requires preparatory thought. Starting from the description of the two problems allegedly caused by problematic definitions – the ‘data problem’ and the ‘problem of ethical pre-emption’ – we performed a logical analysis of how exactly the problems might be caused by definitions. Our approach is based on standards on the theory of definition [23].

(C) To answer how specific definitional content can cause the data or pre-emption problem, we linked the results of (A) to distinctions made concerning topic (B). For each of the definitional categories, we discussed how it might contribute to the ‘data problem’ and the ‘problem of ethical pre-emption’ in the ways distinguished in (B).

## Results

### (A) systematic search for guideline definitions and decomposition

We included 31 publications. Most publications are from Europe or the US/Canada. One publication is from Japan and one from Australia and New Zealand. The guidelines are mostly authored by national palliative care societies in Europe, or more regional palliative care programs in the US and Canada. Two publications are authored by European medical societies. See Table 3 (See Additional file 3 for the landscape format of the Table). One guideline [52] did not have any identifiable definition. Authors of one other guideline are indecisive about the terminology [53]. We excluded these publications, leaving 29 publications for from further analysis. Also, we found a newer guideline from Japan [54] that is available in Japanese only and used the older translation by [50] because of that.

**Table 3:** Extracted definitions with deconstruction of content, sorted by year of publication (* = our translation)

Guideline	Definition	Action	Intended path of action	Intention (purpose)	Unintended consequence	Means	Object	Moral property
Waterloo Wellington Local Health Integration Network (Canada) [24], 2019	“Palliative Sedation Therapy (PST) is the intentional induction and continuous maintenance of a reduced level of consciousness to relieve a patient’s refractory symptom(s) during their last days and weeks of life.”	intentional induction and continuous maintenance of a reduced level of consciousness		relieve refractory symptoms			patients in their last days and weeks of life	
French National Authority for Health (France) [25], 2018	“Palliative sedation seeks, through use of medicines, to reduce alertness, which can lead to loss of awareness. Its goal is to reduce or eliminate the perception of a situation seen as intolerable by the patient, when all the other means available and suitable for the situation have been proposed and/or implemented, without providing the expected relief. The sedation can be intermittent, transient or continuous.”	use of medications	reduce alertness (which can lead to loss or awareness and which can be intermittent, transient or continuous)	reduce or eliminate the perception of a patient, when all the other means available and suitable for the situation have been proposed and/or implemented, without providing the expected relief		medicines	patients perceiving a situation as intolerable	
Alberta Health Services (Canada) [26], 2018	“For the purpose of this Clinical Knowledge Topic (CKT), palliative sedation is the process of inducing and maintaining deep sleep, in the final hours to days of life, for the relief of severe suffering caused by one or more intractable symptoms when all appropriate alternative interventions have failed to bring adequate symptom relief.”	inducing and maintaining deep sleep		the relief of severe suffering caused by one or more intractable symptoms when all appropriate alternative interventions have failed to bring adequate symptom relief			in the final hours to days of life	
Champlain Hospice Palliative Care Program (USA) [27], 2018	“Palliative sedation therapy: [then quote of the EAPC definition].”	(see EAPC)	(see EAPC)	(see EAPC)		(see EAPC)		(see EAPC)
Winnipeg Regional Health Authority (Canada) [28], 2017	“Sedation for Palliative Purposes is the planned and proportionate use of sedation to reduce consciousness in an imminently dying patient with the goal to relieve suffering that is intolerable to the patient and refractory to interventions that are acceptable to the patient.”	planned and proportionate use of sedation	reduce consciousness	relieve suffering that is intolerable to the patient and refractory to interventions that are acceptable to the patient			imminently dying	
British Columbia Centre for Palliative Care (Canada) [29], 2017	“Palliative Sedation Therapy (PST): The monitored use of pharmacological agent(s) to intentionally reduce consciousness to treat refractory, intractable and intolerable symptoms for a patient at end of life with advanced life-limiting, progressive illness.”	monitored use of pharmacological agents	intentional reduction of consciousness	treat refractory, intractable and intolerable symptoms		pharmacological agents	patients at the end of life with advanced life-limiting, progressive illness	
The Australian and New Zealand Society of Palliative Medicine (Australia and New Zealand) [30], 2017	“Palliative Sedation Therapy (PST) is the monitored use of medications to lower a patient’s awareness in order to provide relief of symptoms that are refractory to usual measures, are distressing and result in considerable suffering if unrelieved [.]”	monitored use of medications	lower a patient’s consciousness	relief of symptoms that are refractory to unusual measures, distressing and result in considerable suffering if unrelieved		medications		
Austrian Palliative Society (Austria) [31], 2017*	In the context of palliative medicine, therapeutic (or palliative) sedation is understood as the monitored use of medications with the aim of reducing or eliminating the state of consciousness (unconsciousness), in order to relief the burden of symptoms in an otherwise refractory situation in a manner that is ethically acceptable to the patients, relatives and health-care providers.	the monitored use of medications	reduced or eliminated state of consciousness (unconsciousness)	relief the burden of symptoms in an otherwise refractory situation		medications		in a manner that ethically acceptable to the patients, relatives and health-care providers
Quebec society of doctors of palliative care (Canada) [32], 2016	“‘Palliative sedation’ is defined as the use of sedative medications to relieve refractory symptoms by a reduction in consciousness.”	use of sedative medications	reduction in consciousness	relieve refractory symptoms		sedative medications		
University of California, Los Angeles Hospital System (USA) [33], 2015	“Palliative Sedation is the controlled administration of medications whose primary or secondary effect is to bring about a reduction in patient consciousness, in order to alleviate or at least render tolerable symptoms that have been refractory to standard comprehensive interventions.”	controlled administration of medications…		alleviate or at least render tolerable symptoms that have been refractory to standard comprehensive interventions		medications whose primary or secondary effect is to bring about a reduction in patient consciousness		
Alberta Health Services, Edmonton Zone Palliative Care Program (Canada) [34], 2015	“Palliative Sedation is a process of inducing and maintaining deep sleep in order to relieve refractory symptoms in patients with an anticipated life expectancy of hours to days [.]”	inducing and maintaining deep sleep		relieve refractory symptoms			patients with an anticipated life expectancy of hours to days	
The Norwegian Medical Association (Norway) [35], 2014	“By palliative sedation is meant pharmacological depression of the level of consciousness in order to alleviate suffering that cannot be relieved in any other way.”	pharmacological depression of the level of consciousness		alleviate suffering that cannot be relieved in any other way		pharmaceuticals		
European Society for Medical Oncology Guidelines Working Group (Europe) [36], 2014	“Palliative sedation is a measure of last resort used at the end of life to relieve severe and refractory symptoms. It is carried out by the administration of sedative medications in supervised settings and is aimed at inducing a state of decreased awareness or absent awareness (unconsciousness). The intent of palliative sedation is to relieve the burden of otherwise intolerable suffering for terminally ill patients and to do so in such a manner so as to preserve the moral sensibilities of the patient, medical professionals involved in his or her care, and concerned family and friends.”	administration of sedative medications (in supervised settings)	inducing a state of decreased or absent awareness	relieve the burden of otherwise intolerable suffering		sedative medications	terminally ill patients	carried out in a manner so as to preserve the moral sensibilities of the patient, medical professionals involved in his or her care, and concerned family and friends
Spanish Medical Colleges Organization and Spanish Society for Palliative Care, Spain [37], 2012*	Palliative sedation is the deliberate reduction of consciousness of a sick person by administering medications appropriate for the purpose of avoiding an intense suffering caused by one or more refractory symptoms. It can be carried out continuously or intermittently and with a depth adjusted to achieve the minimal level of sedation in order to relieve the symptom.	administering medications				medications appropriate for the purpose of avoiding an intense suffering caused by one or more refractory symptoms		
Canadian Society of Palliative Care Physicians (CSPCP) Taskforce, Canada [38], 2012	“Many definitions have been put forward for various types of sedation used in palliative practice, but at the core they share the ideas that palliative sedation is: 1) the use of (a) pharmacological agent(s) to reduce consciousness; 2) reserved for treatment of intolerable and refractory symptoms; and 3) only considered in a patient who has been diagnosed with an advanced progressive illness.”	the use of (a) pharmacological agent(s)	reduce consciousness	treatment of intolerable and refractory symptoms		(a) pharmacological agent(s)	patients diagnosed with an advanced progressive illness	
Flanders Federation for Palliative Care (Belgium) [39], 2012*	Definition ‘palliative sedation’: The administration of sedatives in dosages and combinations required to reduce the consciousness of a terminally ill patient as much as needed to control one or more refractory symptoms in an adequate manner.	administration of sedatives				sedatives in dosages and combinations required to reduce the consciousness of a terminally ill patient as much as needed to control one or more refractory symptoms in an adequate manner		
Irish Association for Palliative Care (Ireland) [40], 2011	“[The EAPC sentence is quoted.] ‘In palliative sedation, the physician intends only to relieve severe refractory suffering using sedation as a last resort. There is no intention to end the patient’s life as in euthanasia and physician-assisted suicide.	(see EAPC)	(see EPAC)	(see EAPC)	other purposes	(see EAPC)		(see EAPC)
Fraser Health Hospice Palliative Care Program (Canada) [41], 2011	“Palliative Sedation Therapy (PST) (also ‘Terminal Sedation’, ‘Controlled Sedation’, ‘Total Sedation’, ‘Deep Sedation’, ‘Continuous Sedation’) is the intentional lowering of a patient’s level of consciousness in the last days of life.”	intentional lowering of a patient’s level of consciousness					patients in their last days of life	
National Hospice and Palliative Care Organization (USA) [42], 2010	“Palliative sedation is the lowering of patient consciousness using medications for the express purpose of limiting patient awareness of suffering that is intractable and intolerable.”	lowering of patient consciousness		limiting patient awareness of suffering that is intractable and intolerable		medications		
Swedish Medical Association (Sweden) [43], 2010*	“In this context, palliative sedation means the deliberate lowering of a patient’s consciousness at the end of life with the purpose to achieve a relief from intractable symptoms. The treatment presupposes that the expected lifespan is very short, usually one to two weeks. The intention of palliative sedation is neither to shorten nor to prolong the dying process.”	deliberate lowering of a patient’s consciousness		relief from intractable symptoms	neither shorten or prolong dying process		patients at the end of life with very limited lifespan (usually one to two weeks)	
European Association for Palliative Care (Europe) [15], 2009	“Therapeutic (or palliative) sedation in the context of palliative medicine is the monitored use of medications intended to induce a state of decreased or absent awareness (unconsciousness) in order to relieve the burden of otherwise intractable suffering in a manner that is ethically acceptable to the patient, family and health-care providers.”	monitored use of medications	induce a state of decreased or absent awareness (unconsciousness)	relieve the burden of otherwise intractable suffering		medications		carried out in a manner that is ethically acceptable to the patient, family and health-care providers
Royal Dutch Medical Association (The Netherlands) [44], 2009	“Palliative sedation is defined by the committee as: *The deliberate lowering of a patient’s level of consciousness in the last stages of life.*”	the deliberate lowering of a patient’s level of consciousness					patients in the last stages of life	
French Society of Support and Palliative Care (France) [45], 2009*	Definition of sedation: Sedation is the attempt to reduce vigilance which can go as far as to the loss of consciousness. The goal is to reduce or remove the perception of a situation that is experienced as unbearable by a patient, after all means available and adequate in this situation have been proposed and/or administered without giving the desired alleviation. Sedation (…) can be applied intermittently, transitorily or continuously.	the attempt to reduce vigilance (which can go as far as to the loss of consciousness and which can be applied intermittently, transitorily or continuously)		reduce or remoFrench National Authority for Healthve the patient’s perception of a situation (after all means available and adequate in this situation have been proposed and/or administered without giving the desired alleviation)			patients experiencing their situation as unbearable	
Veterans Health Administration (USA) [46], 2007	“For purposes of this analysis, the NEC defines palliative sedation as: The administration of nonopioid drugs to sedate a terminally ill patient to unconsciousness as an intervention of last resort to treat severe, refractory pain or other clinical symptoms that have not been relieved by aggressive, symptom-specific palliation.”	the administration of nonopioid drugs	sedate to unconsciousness	treat severe, refractory pain or other clinical symptoms that have not been relieved by aggressive, symptom-specific palliation (i.e. as a last resort)		nonopioid drugs	terminally ill patients	
Italian Society of Palliative Care (Italy) [47], 2007*	For terminal/palliative sedation so far there has been the agreement: ‘The intentional reduction of consciousness by pharmacological means until loss of consciousness for the purpose of reducing or abolishing the perception of a symptom otherwise intolerable for the patient, despite the use of adequate means to control the symptom, that thus remained refractory.’ […] In this document, the term terminal/palliative sedation is reserved to […] the final phase of life.	the intentional reduction of consciousness until loss of consciousness		reducing or abolishing the perception of a symptom otherwise intolerable for the patient, despite the use of adequate means to control the symptom, that thus remained refractory			patients in the final phase of life	
Italian Society of Neurology (Italy) [48], 2007	“By ‘palliative sedation’, we mean an intentional reduction of vigilance by pharmacological means up to the point of the complete loss of consciousness with the aim of reducing or abolishing the perception of a symptom that would otherwise be intolerable for the patient despite the implementation of the most adequate means aimed at controlling the symptom itself, which is therefore to be considered refractory[.]”	intentional reduction of vigilance up to the point of the complete loss of consciousness		reducing or abolishing the perception of a symptom that would otherwise be intolerable for the patient despite the implementation of the most adequate means aimed at controlling the symptom itself, which is therefore to be considered refractory				
Swiss Society for Palliative Medicine, Care and (Switzerland) [49], 2005*	Definition of palliative sedation: Deliberate administration of sedating medications, in the smallest effective dosage, in close collaboration with a competent interdisciplinary team, to sustainably relieve (easily evaluable goals have to be formulated), one or more refractory symptoms, of a patient with an advanced illness and limited life expectancy (days, weeks), by permanently or temporarily reducing the patient’s consciousness.	deliberate administration of medications in close collaboration with a competent interdisciplinary team	reducing the patient’s consciousness permanently or temporarily	relieve one or more refractory symptoms sustainably		sedating medications, in the smallest effective dosage		
Japanese Society for Palliative Care (Japan) [50], 2005	“Palliative sedation therapy is defined as (1) the use of sedative medications to relieve suffering by the reduction in patient consciousness level or (2) intentional maintenance of reduction in patient consciousness level resulting from symptomatic treatments.”	use of sedative medications	(1) reduction in patient consciousness level or (2) intentional maintenance of reduction in patient consciousness level resulting from symptomatic treatments	relieve suffering		[for (1):] sedative medications		
Hospice & Palliative Care Federation of Massachusetts (USA) [51], 2004	“*Palliative Sedation* is the monitored use of medications (sedatives, barbiturates,neuroleptics, hypnotics, benzodiazepines or anesthetic medication) to relieve refractory and unendurable physical, spiritual, and/or psychosocial distress for patients with a terminal diagnosis, by inducing varied degrees of unconsciousness. The purpose of the medication(s) is to provide comfort and relieve suffering and not to hasten death.”	monitored use of medications	varied degrees of unconsciousness	relieve refractory and unendurable physical, spiritual, and/or psychosocial distress	hastening death	medications (sedatives, barbiturates,neuroleptics, hypnotics, benzodiazepines or anesthetic medication)	patients with a terminal diagnosis	

We had problems with extracting definitions. The sentences we extracted were not marked as a definition in some guidelines [15, 26, 31, 35, 36, 38, 40], leaving it to the reader whether to take them as full-fledged definitions. In some cases it was not clear where the authors intended their definition to end [40–43, 47]. This was problematic, especially when several sentences are given after a headline “definition” that might not all belong to the definition [40, 43, 47, 51]. A further challenge to identify definitions was inconsistent reference from one guideline to another regarding definition. A guideline from France, for example, referred to a definition in an older French guideline, but the translation and the original differ significantly (cf. [25] and [45]). Two guidelines from Italian medical societies published in 2007 (cf. [47] and [48]) lso give different definitions, though they might have been intended as verbal translations of each other. The guideline from Austria refers to the EAPC definition but does not translate it precisely (cf. [31] and [15]). The term to be defined varied slightly. “Palliative sedation” was used most frequently, sometimes stated with a synonym, for example, “therapeutic sedation”. Table 3 shows all the definitions and content for each category. Please note that the same category can be used with a different content. We will summarise the categories descriptively below and refer to Table 3 when giving examples:

**Content in the category “Action”** As definitional content in this category, “administering medications” or “reduction of consciousness” is used as content here with several variations in detail. Using “administering medications” (or similar) renders the definition dependent on one procedure. “Reduction of consciousness”, however, allows different means of sedation, which could, in theory, also be non-medicamentous (e.g. hypnosis with the result of a light and intermittent change in the level of consciousness).

**Content in the category “Intention (purpose)”** A change in the ‘target’ of the action is stated in this category: for some, it is the perception of the patients [37, 40, 42, 45], for others, it is pain, symptoms or suffering. It is notable that the publication from Austria, where the definition refers to the EAPC definition translates “relieve the burden of otherwise intractable suffering” as “[reduzieren] der Symptomlast in anderweitig therapierefraktären Situationen” [31], meaning “reduce the burden of symptoms in otherwise intractable situations”. In two cases, where an intention was not formulated ([37, 39], cf. Table 3), the means was specified as being ‘adequate for a purpose’ instead. No purpose (and not even this adequacy for a purpose of the means mentioned before) was given at all in two cases [41, 44].

**Content in the category “Intended path of action”** Some definitions state a purpose and the intended route of accomplishing it (see Table 3). A simplified example would be “administering medications (action) ‘in order to’ relieve suffering (purpose) ‘by’ reduction of consciousness (intended path of action)”. This category is the place to state changes in consciousness (vigilance) when not already stated in the category of action. A basic difference is whether unconsciousness is merely an option (e.g.[24] or logically necessary [46].

**Content in the category “Unintended consequence”** Here, either exclusiveness of a given purpose is stated [40] or hastening death is excluded as a purpose of the practice [43, 51]. When purposes are excluded, in each case, the relevant passage in the guideline consists of several sentences that might or might not all belong to the definition. Consequently, it is not clear whether the unintended consequence really belongs to the definition. It might also be additional information.

**Content in the category “Means”** There is considerable variance regarding whether to use this definitional element or not. When used, definitions differ regarding whether to specify medications further up to specific pharmacological classes [46, 51] or to remain unspecific (e.g. “medications”, see Table 3). As mentioned regarding the category “Intention (purpose)”, one noticeable, although rare, strategy is to specify medications concerning their appropriateness for a purpose [37, 39].

**Content in the category “Object”** Patients and their conditions are described with content in this definitional category. When this category is used, the descriptions range from “merely” an advanced life-limiting illness to imminence of death. It is possible regarding the content in this category to narrow the definition down, for example, to patients with a certain life expectancy. However, we would like to emphasise for better understanding, that not specifying the patient’s condition in the definition at all does not imply permissiveness of the practice for all patients. It merely implies that the ‘designation of the treatment’ does not depend on, for example, life expectancy.

**Content in the category “Moral property”** Here, the practice, if used at all, is described as morally right for a list of stakeholders, such as patients and relatives. Adding explicit moral evaluations to the definition is a rarely used strategy but, nonetheless, an important one, since it is used by the EAPC and, thus, in all terminological references to the EAPC [27, 31, 40]. The only other publication using content in this category has the same first author [36].

### (B) possible contributions of definitions to ‘data problem’ and ‘problem of ethical pre-emption’

Independently of the results in (A) and based on standard from theory of definition [23], we concluded that at least three logically possible ways in which definitions might cause the ‘data problem’ and two possible ways to cause the ‘pre-emption problem’ can be distinguished:

**(I) Different definitions: two (or more) different definitions applied to similar cases.** If empirical studies use different definitions, then data will probably (though not necessarily) be different. Exemplarily, if the definition used in one study includes intermittent and/or mild sedation while such practices are excluded by the definition in another study, then the frequency reported will differ.

**(II) Deviating implicit concepts: one definition, but two (or more) implicit understandings of definitional parts applied to similar cases.** This problem can persist ‘even if there is only one operational definition and even if it is precise’. One clinician, for example, might tie the meaning of “palliative sedation” implicitly to deep and continuous sedation of patients until death even if the definition used in the study also includes practices of mild and intermittent sedation.

**(III) Disagreement on the facts: one definition but different opinions about the fulfillment of definitional criteria.** If one of the definitional criteria is difficult to assess, a matter of feeling or can only be estimated imprecisely, then, as the third possible way, consensus about how to label a case will be difficult to achieve. Thus, labelling might be inconsistent. One example is the use of the criterion “severe suffering”. If two clinicians disagree whether a patient prior to administration of a sedative was “suffering severely”, then they may disagree about whether the case should be labelled as one of “palliative sedation”. Same holds true for “in the last days of life”. Therefore, even if they follow the same and precise definition in a strict way and have the same implicit understanding of the terms, they might label cases differently.

Adding to these ways of causing data problems, at least two ways should be distinguished that might lead to a concern of ethical pre-emption, i.e. concerns about pre-empting critical ethical discussion of the practice by reference to its definition:

**(IV) Explicit normativity** A definition of sedation in palliative care can be explicitly normative, in the sense that cases are only included that are assessed to be “good practice”. The EAPC definition [15] can be read in such a way. Those who are concerned about bad practice or have a more critical perspective of the practice might find it difficult to even raise their criticism. Their fear (and it might happen) that their concerns are said to be ruled out ‘by definition.’

**(V) Implicit normativity** A definition is implicitly normative if parts of it are justified by moral arguments. In this way, the scope of the sedation practice is narrowed down indirectly to usage that is deemed to be legitimate. An example is adding “refractory” to the definition, when sedating in situations of ‘non’-refractory symptoms is deemed to be problematic from a moral perspective. Again, critics might find it difficult to formulate their disagreement with the practice when it is implicitly narrowed down to legitimate use.

### (C) how content of the definitions contributes to the ‘data’ and ‘pre-emption’ problem in different ways

Having ‘deconstructed’ the definitions in the guidelines, we can link them to the five ways through which the two problems reported (concerning data and ethical pre-emption) might be caused and, thus, bring together results of (A) and (B). In a first step, we demonstrate contributions to the ‘data problem’ and in a second step, contributions to the ‘problem of ethical pre-emption’.

#### How guideline definitions contribute to the ‘data problem’

##### Possible contributions by the category “Action”

At first sight, content here can reduce the problem of **disagreement about the facts** (III) by clarifying what is done. “Reduction of consciousness”, as one possible content in this category, however, might require specification of depth (i.e. deep, mild) and mode (i.e. intermittent, continuous). This may also result in a specification of subtypes of the defined practice. Otherwise – a problem of deviating implicit concepts (II) – different types of sedation might be conflated. In addition, content in this category can generate **disagreement about the facts** (III). One example would be a clinical situation in which there is doubt whether a “reduction of consciousness” was a result of medication or the consequence of the natural course of disease.

##### Possible contributions by the category “Intention (purpose)”

Defining by intention has been suggested as the main cause of conceptual confusion [8, 55]. However, it should be noted that “intention” can be understood in at least two ways: ‘psychologically’, “intention” refers to a state of mind; ‘pragmatically’, it refers to features of the treatment. Understood psychologically, intentions are controversially discussed [9, p. 428f.], [56], since agreement about the intentions involved might be difficult to achieve and **disagreement about the facts** (III) might be the consequence. In the second (pragmatic) meaning it is understood that the intention is independent of the state of mind as long as the actions carried out are the ones one ‘would have chosen if’ one followed the intention. One example of making use of this understanding of intention is the distinction of euthanasia from sedation practices with reference to a specific pattern of practice: “The difference hinges upon whether or not the medications are being titrated to effect. […] If you are titrating to comfort, you are not intending the patient’s death” [57, p. 59].

Empirical reports of mixed and problematic intentions, for example, self-reported intentions of physicians to hasten death when performing sedation [58, p. 182], should only cause conceptual concerns when “intention” is understood psychologically. Therefore, distinguishing the two concepts of intention is important. Using one concept in one situation and the other one in the next might result in data problems via **deviating implicit concepts** (II).

The targets of the practice stated in this category often include refractoriness. This content of the definitional cetegory is prone to **disagreement about the facts** (III), since refractoriness might be assessed controversially in a clinical situation. The same holds true for the decision on whether suffering is “unbearable” [28, 29, 36, 38, 42, 47, 48, 51].

##### Possible contributions by the category “Intended path of action”

The intention here can also be interpreted psychologically or pragmatically, with the same possible effects. Has the path to be present “mentally” or does it depend on the specific clinical actions carried out?

##### Possible contributions by the category “Non-intendend consequences”

Again, all comments regarding “intention” also generally apply here. Assume, for example, that hastening death is explicitly excluded as a purpose [51]. Then, in cases where clinicians accepted or would have appreciated a life-shortening effect, they might consider this part of the definition as not fulfilled (psychological interpretation) or, nonetheless, fulfilled (pragmatic) when treatment was carried out properly. Mixing psychological and pragmatic readings will result in **deviating implicit concepts** (II). Psychological meaning will probably lead to **disagreement about the facts** (III), since intentions have many facets psychologically that hamper simple agreement on who had which intentions [56].

##### Possible contributions by the category “Means”

Using content in the definition that specifies the means can have the result that the defined practice becomes outdated “technologically”, at least in principle. For sedation practice this might happen when interventions other than pharmacological ones become plausible means. This could result in **different definitions** (I) in study comparison. However, this risk is currently low, since only hypnosis is the other intervention candidate and, so far, only at the lower end of the spectrum of sedative effects (mild and intermittent).

The same seems to hold true for **deviating implicit concepts** (II): as long as interventions are almost exclusively pharmacological, the risk of implicit different interpretations is low. However, the risk increases with formulations that might be interpreted as different classes of substances by different interpreters. Do “sedatives”, for example, include opiates because of their sedative effect in higher dosages or only medications that are approbated primarily for their sedative effects, such as benzodiazepines and anaesthetics? Implicit deviations influencing quantitative data might be a result.

##### Possible contributions by the category “Object”

This category brings a high risk of all types of causes of data problems, since it specifies the patients treated. First of all, and obviously: studies limited to a specific patient population should not be compared with studies that did not specify patient condition or specified it differently, because they use different definitions. This includes especially definitional content specifying life expectancy [24, 26, 28, 29, 34, 36, 41, 43, 44, 46, 47, 51].

Great attention should be paid to **deviating implicit concepts** (II): even if the definition is well-made and precise, it is easy to imagine that patients are included in statistical analysis who violate the definition (e.g. a patient with unclear or longer life prognosis in a clinical crisis). Much depends on whether those who “apply” the definition treat information about patient condition as a strict inclusion criterion or as a noncommittal description of “typical” patients.

**Disagreement on the facts** (III) is to be expected, since typical content in this category, such as life expectancy, is not easy to assess. The assessment of identical cases might vary between practitioners and wards, with varying labelling of cases and, thus, varying statistical reports.

##### Possible contributions by the category “Moral properties”

Adding content here can pose challenges for research because it is likely that there will be different moral evaluations of a specific case and, thus, **disagreement about the facts** (III) – in this case, on the moral value of the measure. There might, for example, be disagreement about whether sedation was indeed acceptable for the family of the patient. Moreover, a definition encompassing a moral property cannot be used to operationalise the object of a study when the study’s goal is to survey a range of practices broadly. This is because the use of the category automatically narrows the ranges of practices elicited down to those deemed to be morally acceptable. Only cases which are in line with the ethical requirements would be included. Not paying attention to this logical consequence could result in **different definitions** (I) in study comparison.

#### Results concerning the ‘problem of ethical pre-emption’

##### Possible contributions by normativity of definitional content

Regarding implicit normativity (as defined above), we see issues of perspective on and “handling” of definitions rather than a problem of specific definitional content. Content in all categories can be seen as implicitly normative: use of the respective category and decision regarding a specific content might be motivated by ethical considerations. As an example, “monitoring” or “medications” as a means [15] is stated because it is considered to be the state-of-the-art of ethical practice to only use medication or to monitor patients.

Based on our analysis, we conclude that this is the case for content in every category. Obvious examples are definitional contents regarding intractability of symptoms, explicit formulations of life expectancy or unintended consequences. Such content can be found in all guideline definitions, even in those which are intentionally formulated in a more “descriptive” way. Why, for example, does the guideline definition from the Netherlands include “in the last stage of life” in its definition [44, 59]? It is because the committee holds sedation for symptom control to be ‘acceptable’ under this (vague) condition: “Lowering the patient’s consciousness to relieve suffering is appropriate in the last stages of life, in which death is expected to ensue in the near future” [44, p. 19]. One purpose of choosing a definition thus seems to be to express the ethical considerations in the guideline regarding good practice.

As an example, the definition in the EAPC guideline would, in this respect, not be intended to “neutrally” describe the practice to be regulated with the guideline. Instead, the clinical activity to be regulated, is – as stated in the title – “the use of sedation in palliative care”. Accordingly, the document is the result of the EAPC’s considerations about rules of good practice for this activity. Parts of these considerations are summarised and expressed in a definition of a specific medical practice termed “palliative (or therapeutic) sedation”. We do not see how any normativity would, in fact, imply a ‘problem of ethical pre-emption’ that requires solutions when this is considered. However, it is important to be able to formulate ethical concerns about a practice. We will discuss strategies to accomplish this below.

## Discussion

The preceding analyses indicate that common ground regarding definitions of sedation in palliative care is small. In fact, “reduced consciousness” (or similar) is the only content shared by all definitions analysed. This finding contrasts with the statement in the latest systematic review on guidelines, according to which “Palliative sedation was defined in analogous ways in all guidelines, that is, as an intervention instituted solely for the purpose of refractory symptom control” [60, p. 225]. However, as shown in our analysis, stating an intention does not form part of all guideline definitions. In addition, guidelines often refer to the relieving of “suffering” but not of “symptoms” and rarely exclude purposes explicitly.

As our results show, search for a ‘point of convergence’ of definitions won’t lead to well-build terminology. Because of that we suggest options for systematic conceptual improvement in the following and no statistical argument (‘The more often used, the better the definition’).

### Improving in light of the ‘data problem’

Revisiting **different definitions** (I) as the first possible cause of the ‘data problem’, an obvious solution is the use of only one precise definition in a study. This becomes even more important in study comparison. Thus, respective publications should provide detailed information about the types of sedation (i.e. mode and depth) included and all other exclusion and inclusion criteria. This could relieve a considerable part of the uncertainty and would be in line with repeated pleas to solve existing inconsistencies by uniform definitions [61, p. 310] or by defining subcate- gories of a broad definition [62, p. 447].

A clearer picture of the definitional elements that should be used and reported as part of the research can be achieved by considering strategies regarding the two other possible ways to cause the ‘data problem’. Concerning **deviating implicit concepts** (II), improvement seems possible by providing definitions of the defining terms or by adding clarifying comments. One possible improvement (for the category of “Intended path of action”) would be defining “reduced consciousness” explicitly. This could be done by using scales, such as RASS [63], RASS-PAL [64] or other assessment techniques. A second option (to clarify the category of “moral properties”) would be handing out additional information on what is meant by “ethically acceptable” to guide on the relevance of ethical evaluation. Thirdly, regarding the formulation of means, pharmacological accuracy is needed (e.g. specify “sedative” [32, 36, 39, 49, 50]). A fourth option to reduce deviating implicit concepts would be to clarify the category of “object”. One might clarify, for example, “terminally ill patients” by pointing out that this covers patients with a prognosis of a maximum of 14 days according to the treating physician. One additional chance is to clarify the meaning of the category “intention” – if it is used as part of the definition at all. In this respect, the definitions from Spain and Belgium [37, 39] are examples of how to express a clearly “pragmatic” understanding of intention, e.g. “sedatives in dosages [dots] *required to* reduce the consciousness […] *as much as needed to* control one or more refractory symptoms in an adequate manner” [39] (our emphasis).

One tool that can be utilised as a strategy to identify implicit concepts can be cognitive interviews with participating researchers to identify their implicit (mis)understandings of a definition before using it [65]. Such interviews could be informed by our analysis of definitional categories.

Concerning the third way to cause the ‘data problem’ **disagreement about the facts** (III), our analysis shows that several expressions are particularly prone to such a challenge, but for different reasons. One source can be areas in which it is known that professional judgments are uncertain and differ, for example, concerning the prognosis of life expectancy in the category of object or intractability in the category of purpose. Another reason for disagreement about facts are judgments about mental states involved in “unbearable suffering” and in the category of purpose. In addition, formulations of ethical properties are prone to disagreement about facts due to moral pluralism in societies. Identifying and discarding those elements from the definition that are prone to disagreement should be considered.

A systematically constructed new proposal of terminology is beyond the scope of this article. Nevertheless, answering the following question should improve the terminological quality of future definitions:

1. Have you discussed each definitional element for implications and alternative formulations?
2. Is the definitional element necessary and have you relocated nice-to-have information to supplementary material?
3. Can you be sure that readers interpret the definition consistently? If not, can you improve this by reformulation or supplementary information?
4. Have you minimised the risk of disagreement about facts for each category?
5. Have you tested the reliability of the definition?

One promising option in the context of guidelines can be refraining from the definition of the good practice measure in favour of a precise definition of “sedation”, accompanied by rules of good practice for “sedation in palliative care” – not because of concerns about pre-emption but for the goal of avoiding confusion, implicit assumptions and unsound data. Assessing the pros and cons of this option requires interdisciplinary research, which, in addition to conceptual analyses, should also include empirical (qualitative) data on the use of concepts of sedation, misunderstandings and associations, as well as legal requirements for definitions.

### Improving in light of the ‘problem of ethical preemption’

Our analyses indicate that some elements of the definitions are obviously the result of ethical considerations about due care. There is no ‘problem of ethical pre-emption’ when discourse partners accept this and argue accordingly.

Distinguishing the purpose of a definition in a guideline from the purpose of a definition that responds to requirements of empirical research is important to reduce the current confusion in palliative sedation discourse. A possible (though not necessary) requirement for research purposes is that the definition covers a wide scope of practices, regardless of whether they fit an ideal of good practice. By contrast, a definition of a practice in a guideline often (though not necessarily) focuses on normative criteria which automatically limit its scope. Against this background, the use of guideline definitions in empirical work which aims to explore sedation practices generally often seems ill-informed. Moreover, distinguishing purposes of definitions solves the ‘problem of ethical pre-emption’ mentioned above, since it makes explicit that a guideline definition often does not serve the purpose of describing a practice but often aims to express a regulated practice. The use of positive moral aspects in a definition of sedation practices has even been called “intellectually dishonest”[9, p. 429]. Unsurprisingly, the authors attacked responded stridently [66, p. e11 f.]. Such a general discussion about neutrality or normativity of definitions without recognising different purposes of definitions seems pointless to us.

Instead, ethical concerns regarding, for example, indications, motivation and medications used [1] can all be formulated referring to the use of ‘sedation in palliative care’ – regardless of the definitions in guidelines. Conversely, criticism of the practice or regulations of ‘sedation in palliative care’ cannot be answered by referring to a definition.

### Limitations

The systematic literature search has some limitations and a possible bias towards English speaking countries. Potential further publications not written or tagged in English might reveal additional definitional strategies. The same might be true for legal documents, palliative care text books or single hospital documents. Interpretative misunderstandings during extraction and translation of the definitions might are also possible. As mentioned above, we had to interpret passages of some publications to identify a definition. This may have influenced our analysis. It also indicates though, that core terminology should be easier to identify in guidelines.

## Conclusion

There is a lack of consensus and a high potential even for different kinds of confusion regarding the labeling of sedation practices in palliative care. Separate solution strategies can be formulated for these different kinds of problems. Calling for uniformity of definitions alone, without an understanding of the underlying types of problems, will not help to improve the conceptual situation concerning sedation in palliative care. Instead, the categories presented and our analyses of impact on conceptual problems in different ways can serve as a starting point when constructing terminology. They can guide reflection on the intuitive use of terms and be used to explore whether concepts are confused in communication in research or everyday practice. In addition, our methodological distinction of different purposes of the definition and implications may further the dissent on pre-emption of the ethical dispute about sedation practices in palliative care.

## Additional material

Additional file 1 PRISMA Flow diagram.

Additional file 2 PRISMA checklist.

Additional file 3 Extracted definitions with deconstruction of content, sorted by year of publication (* = our translation)

## Data Availability

Exact search strategy and initial search results of the systematic literature search can be obtained from the authors.
