# Supplementary material for: What do you mean by “palliative sedation”? Pre-explicative analyses as preliminary steps towards better definitions
Source: BMC Palliat Care. 2020 Sep 23;19:147. doi: 10.1186/s12904-020-00635-9 (PMC7513316; doi:10.1186/s12904-020-00635-9)
Supplement: Supplementary file 1 — PRISMA Flow diagram. [file 12904_2020_635_MOESM1_ESM.pdf]

PRISMA 2009 Flow Diagram

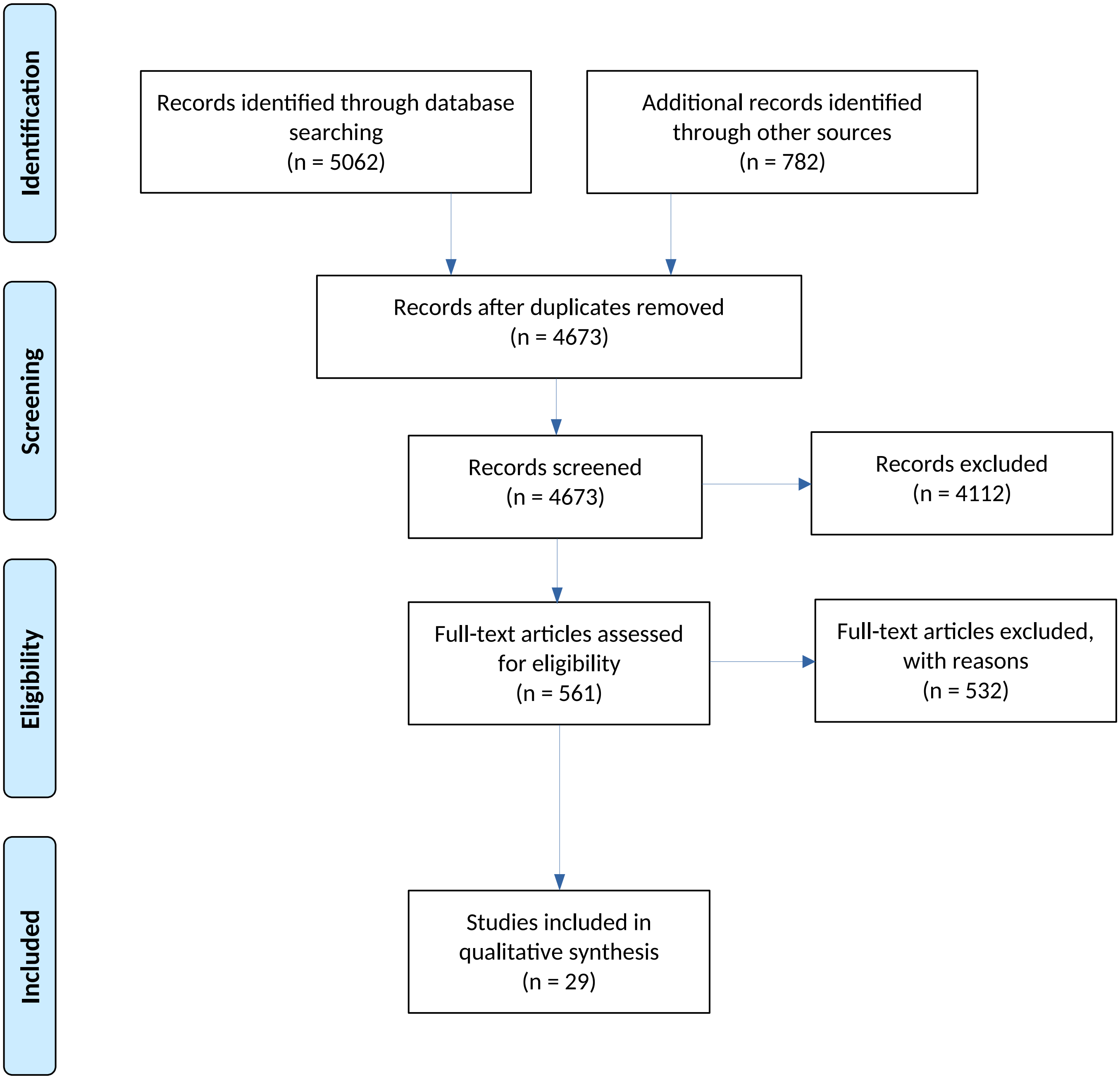

From: Moher D, Liberati A, Tetzlaff J, Altman DG, The PRISMA Group (2009). *P*referred *R*eporting *I*tems for *S*ystematic Reviews and *M*eta-*A*nalyses: The PRISMA Statement. PLoS Med 6(7): e1000097. doi:10.1371/journal.pmed1000097

For more information, visit www.prisma-statement.org.
