# Supplementary material for: What do you mean by “palliative sedation”? Pre-explicative analyses as preliminary steps towards better definitions
Source: BMC Palliat Care. 2020 Sep 23;19:147. doi: 10.1186/s12904-020-00635-9 (PMC7513316; doi:10.1186/s12904-020-00635-9)
Supplement: Supplementary file 3 — Extracted definitions with deconstruction of content, sorted by year of publication (* = our translation) [file 12904_2020_635_MOESM3_ESM.pdf]

Extracted definitions with deconstruction of content, sorted by year of publication (* = our translation)

Guideline	Definition	Action	Intended path of action	Intention (purpose)	Unintended consequence	Means	Object	Moral property
Waterloo Wellington Local Health Integration Network (Canada) [24], 2019	"Palliative Sedation Therapy (PST) is the intentional induction and continuous maintenance of a reduced level of consciousness to relieve a patient's refractory symptom(s) during their last days and weeks of life."	intentional induction and continuous maintenance of a reduced level of consciousness		relieve refractory symptoms			patients in their last days and weeks of life	
French National Authority for Health (France) [25], 2018	"Palliative sedation seeks, through use of medicines, to reduce alertness, which can lead to loss of awareness. Its goal is to reduce or eliminate the perception of a situation seen as intolerable by the patient, when all the other means available and suitable for the situation have been proposed and/or implemented, without providing the expected relief. The sedation can be intermittent, transient or continuous."	use of medications	reduce alertness (which can lead to loss or awareness and which can be intermittent, transient or continuous)	reduce or eliminate the perception of a patient, when all the other means available and suitable for the situation have been proposed and/or implemented, without providing the expected relief		medicines	patients perceiving a situation as intolerable	
Alberta Health Services (Canada) [26], 2018	"For the purpose of this Clinical Knowledge Topic (CKT), palliative sedation is the process of inducing and maintaining deep sleep, in the final hours to days of life, for the relief of severe suffering caused by one or more intractable symptoms when all appropriate alternative interventions have failed to bring adequate symptom relief."	inducing and maintaining deep sleep		the relief of severe suffering caused by one or more intractable symptoms when all appropriate alternative interventions have failed to bring adequate symptom relief			in the final hours to days of life	
Champlain Hospice Palliative Care Program (USA) [27], 2018	"Palliative sedation therapy: [then quote of the EAPC definition]."	(see EAPC)	(see EAPC)	(see EAPC)		(see EAPC)		(see EAPC)
Winnipeg Regional Health Authority (Canada) [28], 2017	"Sedation for Palliative Purposes is the planned and proportionate use of sedation to reduce consciousness in an imminently dying patient with the goal to relieve suffering that is intolerable to the patient and refractory to interventions that are acceptable to the patient."	planned and proportionate use of sedation	reduce consciousness	relieve suffering that is intolerable to the patient and refractory to interventions that are acceptable to the patient			imminently dying	
British Columbia Centre for Palliative Care (Canada) [29], 2017	"Palliative Sedation Therapy (PST): The monitored use of pharmacological agent(s) to intentionally reduce consciousness to treat refractory, intractable and intolerable symptoms for a patient at end of life with advanced life-limiting, progressive illness."	monitored use of pharmacological agents	intentional reduction of consciousness	treat refractory, intractable and intolerable symptoms		pharmacological agents	patients at the end of life with advanced life-limiting, progressive illness	
The Australian and New Zealand Society of Palliative Medicine (Australia and New Zealand)[30], 2017	"Palliative Sedation Therapy (PST) is the monitored use of medications to lower a patient's awareness in order to provide relief of symptoms that are refractory to usual measures, are distressing and result in considerable suffering if unrelieved [.]"	monitored use of medications	lower a patient's consciousness	relief of symptoms that are refractory to unusual measures, distressing and result in considerable suffering if unrelieved		medications		
Austrian Palliative Society (Austria) [31], 2017*	In the context of palliative medicine, therapeutic (or palliative) sedation is understood as the monitored use of medications with the aim of reducing or eliminating the state of consciousness (unconsciousness), in order to relief the burden of symptoms in an otherwise refractory situation in a manner that is ethically acceptable to the patients, relatives and health-care providers.	the monitored use of medications	reduced or eliminated state of consciousness (unconsciousness)	relief the burden of symptoms in an otherwise refractory situation		medications		in a manner that ethically acceptable to the patients, relatives and health-care providers
Quebec society of doctors of palliative care (Canada) [32], 2016	"'Palliative sedation' is defined as the use of sedative medications to relieve refractory symptoms by a reduction in consciousness."	use of sedative medications	reduction in consciousness	relieve refractory symptoms		sedative medications		
University of California, Los Angeles Hospital System (USA) [33], 2015	"Palliative Sedation is the controlled administration of medications whose primary or secondary effect is to bring about a reduction in patient consciousness, in order to alleviate or at least render tolerable symptoms that have been refractory to standard comprehensive interventions."	controlled administration of medications…		alleviate or at least render tolerable symptoms that have been refractory to standard comprehensive interventions		medications whose primary or secondary effect is to bring about a reduction in patient consciousness		
Alberta Health Services, Edmonton Zone Palliative Care Program (Canada) [34], 2015	"Palliative Sedation is a process of inducing and maintaining deep sleep in order to relieve refractory symptoms in patients with an anticipated life expectancy of hours to days [.]"	inducing and maintaining deep sleep		relieve refractory symptoms			patients with an anticipated life expectancy of hours to days	
The Norwegian Medical Association (Norway) [35], 2014	"By palliative sedation is meant pharmacological depression of the level of consciousness in order to alleviate suffering that cannot be relieved in any other way."	pharmacological depression of the level of consciousness		alleviate suffering that cannot be relieved in any other way		pharmaceuticals		
European Society for Medical Oncology Guidelines Working Group (Europe) [46], 2014	"Palliative sedation is a measure of last resort used at the end of life to relieve severe and refractory symptoms. It is carried out by the administration of sedative medications in supervised settings and is aimed at inducing a state of decreased awareness or absent awareness (unconsciousness). The intent of palliative sedation is to relieve the burden of otherwise intolerable suffering for terminally ill patients and to do so in such a manner so as to preserve the moral sensibilities of the patient, medical professionals involved in his or her care, and concerned family and friends."	administration of sedative medications (in supervised settings)	inducing a state of decreased or absent awareness	relieve the burden of otherwise intolerable suffering		sedative medications	terminally ill patients	carried out in a manner so as to preserve the moral sensibilities of the patient, medical professionals involved in his or her care, and concerned family and friends
Spanish Medical Colleges Organization and Spanish Society for Palliative Care, Spain [47], 2012*	Palliative sedation is the deliberate reduction of consciousness of a sick person by administering medications appropriate for the purpose of avoiding an intense suffering caused by one or more refractory symptoms. It can be carried out continuously or intermittently and with a depth adjusted to achieve the minimal level of sedation in order to relieve the symptom.	administering medications				medications appropriate for the purpose of avoiding an intense suffering caused by one or more refractory symptoms		
Canadian Society of Palliative Care Physicians (CSPCP) Taskforce, Canada [38], 2012	"Many definitions have been put forward for various types of sedation used in palliative practice, but at the core they share the ideas that palliative sedation is: 1) the use of (a) pharmacological agent(s) to reduce consciousness; 2) reserved for treatment of intolerable and refractory symptoms; and 3) only considered in a patient who has been diagnosed with an advanced progressive illness."	the use of (a) pharmacological agent(s)	reduce consciousness	treatment of intolerable and refractory symptoms		(a) pharmacological agent(s)	patients diagnosed with an advanced progressive illness	
Flanders Federation for Palliative Care (Belgium) [39], 2012*	Definition 'palliative sedation': The administration of sedatives in dosages and combinations required to reduce the consciousness of a terminally ill patient as much as needed to control one or more refractory symptoms in an adequate manner.	administration of sedatives				sedatives in dosages and combinations required to reduce the consciousness of a terminally ill patient as much as needed to control one or more refractory symptoms in an adequate manner		
Irish Association for Palliative Care (Ireland) [40], 2011	"[The EAPC sentence is quoted.] 'In palliative sedation, the physician intends only to relieve severe refractory suffering using sedation as a last resort. There is no intention to end the patient's life as in euthanasia and physician-assisted suicide.	(see EAPC)	(see EPAC)	(see EAPC)	other purposes	(see EAPC)		(see EAPC)
Fraser Health Hospice Palliative Care Program (Canada) [41], 2011	"Palliative Sedation Therapy (PST) (also 'Terminal Sedation', 'Controlled Sedation', 'Total Sedation', 'Deep Sedation', 'Continuous Sedation') is the intentional lowering of a patient's level of consciousness in the last days of life."	intentional lowering of a patient's level of consciousness					patients in their last days of life	
National Hospice and Palliative Care Organization (USA) [42], 2010	"Palliative sedation is the lowering of patient consciousness using medications for the express purpose of limiting patient awareness of suffering that is intractable and intolerable."	lowering of patient consciousness		limiting patient awareness of suffering that is intractable and intolerable		medications		
Swedish Medical Association (Sweden) [43], 2010*	"In this context, palliative sedation means the deliberate lowering of a patient's consciousness at the end of life with the purpose to achieve a relief from intractable symptoms. The treatment presupposes that the expected lifespan is very short, usually one to two weeks. The intention of palliative sedation is neither to shorten nor to prolong the dying process."	deliberate lowering of a patient's consciousness		relief from intractable symptoms	neither shorten or prolong dying process		patients at the end of life with very limited lifespan (usually one to two weeks)	
European Association for Palliative Care (Europe) [15], 2009	"Therapeutic (or palliative) sedation in the context of palliative medicine is the monitored use of medications intended to induce a state of decreased or absent awareness (unconsciousness) in order to relieve the burden of otherwise intractable suffering in a manner that is ethically acceptable to the patient, family and health-care providers."	monitored use of medications	induce a state of decreased or absent awareness (unconsciousness)	relieve the burden of otherwise intractable suffering		medications		carried out in a manner that is ethically acceptable to the patient, family and health-care providers
Royal Dutch Medical Association (The Netherlands) [44], 2009	"Palliative sedation is defined by the committee as: *The deliberate lowering of a patient's level of consciousness in the last stages of life.*"	the deliberate lowering of a patient's level of consciousness					patients in the last stages of life	
French Society of Support and Palliative Care (France) [45], 2009*	Definition of sedation: Sedation is the attempt to reduce vigilance which can go as far as to the loss of consciousness. The goal is to reduce or remove the perception of a situation that is experienced as unbearable by a patient, after all means available and adequate in this situation have been proposed and/or administered without giving the desired alleviation. Sedation (…) can be applied intermittently, transitorily or continuously.	the attempt to reduce vigilance (which can go as far as to the loss of consciousness and which can be applied intermittently, transitorily or continuously)		reduce or remoFrench National Authority for Healthve the patient's perception of a situation (after all means available and adequate in this situation have been proposed and/or administered without giving the desired alleviation)			patients experiencing their situation as unbearable	
Veterans Health Administration (USA) [46], 2007	"For purposes of this analysis, the NEC defines palliative sedation as: The administration of nonopioid drugs to sedate a terminally ill patient to unconsciousness as an intervention of last resort to treat severe, refractory pain or other clinical symptoms that have not been relieved by aggressive, symptom-specific palliation."	the administration of nonopioid drugs	sedate to unconsciousness	treat severe, refractory pain or other clinical symptoms that have not been relieved by aggressive, symptom-specific palliation (i.e. as a last resort)		nonopioid drugs	terminally ill patients	
Italian Society of Palliative Care (Italy) [47], 2007*	For terminal/palliative sedation so far there has been the agreement: 'The intentional reduction of consciousness by pharmacological means until loss of consciousness for the purpose of reducing or abolishing the perception of a symptom otherwise intolerable for the patient, despite the use of adequate means to control the symptom, that thus remained refractory.' […] In this document, the term terminal/palliative sedation is reserved to […] the final phase of life.	the intentional reduction of consciousness until loss of consciousness		reducing or abolishing the perception of a symptom otherwise intolerable for the patient, despite the use of adequate means to control the symptom, that thus remained refractory			patients in the final phase of life	
Italian Society of Neurology (Italy) [48], 2007	"By 'palliative sedation', we mean an intentional reduction of vigilance by pharmacological means up to the point of the complete loss of consciousness with the aim of reducing or abolishing the perception of a symptom that would otherwise be intolerable for the patient despite the implementation of the most adequate means aimed at controlling the symptom itself, which is therefore to be considered refractory[.]"	intentional reduction of vigilance up to the point of the complete loss of consciousness		reducing or abolishing the perception of a symptom that would otherwise be intolerable for the patient despite the implementation of the most adequate means aimed at controlling the symptom itself, which is therefore to be considered refractory				
Swiss Society for Palliative Medicine, Care and (Switzerland) [49], 2005*	Definition of palliative sedation: Deliberate administration of sedating medications, in the smallest effective dosage, in close collaboration with a competent interdisciplinary team, to sustainably relieve (easily evaluable goals have to be formulated), one or more refractory symptoms, of a patient with an advanced illness and limited life expectancy (days, weeks), by permanently or temporarily reducing the patient's consciousness.	deliberate administration of medications in close collaboration with a competent interdisciplinary team	reducing the patient's consciousness permanently or temporarily	relieve one or more refractory symptoms sustainably		sedating medications, in the smallest effective dosage		
Japanese Society for Palliative Care (Japan) [50], 2005	"Palliative sedation therapy is defined as (1) the use of sedative medications to relieve suffering by the reduction in patient consciousness level or (2) intentional maintenance of reduction in patient consciousness level resulting from symptomatic treatments."	use of sedative medications	(1) reduction in patient consciousness level or (2) intentional maintenance of reduction in patient consciousness level resulting from symptomatic treatments	relieve suffering		[for (1):] sedative medications		
Hospice & Palliative Care Federation of Massachusetts (USA) [51], 2004	"*Palliative Sedation* is the monitored use of medications (sedatives, barbiturates,neuroleptics, hypnotics, benzodiazepines or anesthetic medication) to relieve refractory and unendurable physical, spiritual, and/or psychosocial distress for patients with a terminal diagnosis, by inducing varied degrees of unconsciousness. The purpose of the medication(s) is to provide comfort and relieve suffering and not to hasten death."	monitored use of medications	varied degrees of unconsciousness	relieve refractory and unendurable physical, spiritual, and/or psychosocial distress	hastening death	medications (sedatives, barbiturates,neuroleptics, hypnotics, benzodiazepines or anesthetic medication)	patients with a terminal diagnosis	
